# MACROD2 expression predicts response to 5-FU-based chemotherapy in stage III colon cancer

**DOI:** 10.18632/oncotarget.25655

**Published:** 2018-06-29

**Authors:** Evert van den Broek, Sjoerd H. den Uil, Veerle M.H. Coupé, Pien M. Delis-van Diemen, Anne S. Bolijn, Herman Bril, Hein B.A.C. Stockmann, Nicole C.T. van Grieken, Gerrit A. Meijer, Remond J.A. Fijneman

**Affiliations:** ^1^ Department of Pathology, VU University Medical Center, Amsterdam, The Netherlands; ^2^ Department of Surgery, Spaarne Gasthuis, Haarlem, The Netherlands; ^3^ Department of Epidemiology and Biostatistics, VU University Medical Center, Amsterdam, The Netherlands; ^4^ Department of Pathology, Spaarne Gasthuis, Haarlem, The Netherlands; ^5^ Department of Pathology, Netherlands Cancer Institute, Amsterdam, The Netherlands

**Keywords:** colon cancer, disease recurrence, predictive biomarker, MACROD2, response to chemotherapy

## Abstract

**Background:**

Colorectal cancer (CRC) is caused by genetic aberrations. *MACROD2* is commonly involved in somatic focal DNA copy number losses, in more than one-third of CRCs. In this study, we aimed to investigate the association of MACROD2 protein expression with clinical outcome in stage II and stage III colon cancer.

**Methods:**

Tissue microarrays (TMA) containing formalin-fixed paraffin-embedded tissue cores from 386 clinically well-annotated primary stage II and III colon cancers were stained by immunohistochemistry and evaluated for MACROD2 protein expression. Disease-free survival (DFS) analysis was performed to estimate association with clinical outcome.

**Results:**

Loss of nuclear MACROD2 protein expression in epithelial neoplastic cells of stage III microsatellite stable (MSS) colon cancers was associated with poor DFS within the subgroup of 59 patients who received 5-fluorouracil (5-FU)-based adjuvant chemotherapy (p=0.005; HR=3.8, 95% CI 1.4-10.0).

**Conclusion:**

These data indicate that low nuclear expression of MACROD2 is associated with poor prognosis of patients with stage III MSS primary colon cancer who were treated with 5-FU-based adjuvant chemotherapy.

## INTRODUCTION

Colorectal cancer (CRC) has a worldwide incidence of over 1.3 million and is one of the leading causes of cancer-related deaths [1]. In the Western world, approximately one-third of CRC patients will die due to disease progression [2]. To estimate the prognosis of CRC patients, tumors are currently classified into stage I to IV according to the tumor-node-metastasis (TNM) staging, which is primarily based upon histopathological features of the tumor. Because somatic DNA alterations enable tumors to progress, characterization of genomic inter-tumor heterogeneity may reveal promising candidate biomarkers that could ultimately improve patient stratification for prognosis and therapy prediction. *MACROD2* has been shown to be commonly affected by focal deletions in CRC genomes [3–5], and has been identified to be the most frequently affected gene by structural variant (SV) breakpoints in CRC [5, 6]. The prevalence of chromosomal breakpoints in *MACROD2* is very high, *i.e.* 41% in a large series of 352 advanced CRC samples [6].

The function of MACROD2 is largely unknown. Recent studies demonstrated that MACROD2 is involved in highly dynamic mono-ADP-ribosylation (MARylation), which is a reversible post-translational protein modification that enables to control functions of target proteins. ADP ribose moieties can be attached to amino acid acceptor sites of target proteins by ADP-ribosyltransferases using the cofactor NAD+. Reversion of this modification is achieved by ADP-ribosylhydrolase activity. The macrodomain containing hydrolase MACROD2 can recognize mono-ADP-ribosyl groups and erase this motif from MARylated proteins. For example, the mono-ADP-ribosylhydrolase activity of MACROD2 is able to restore the WNT inhibitory function of the kinase GSK3B that is modified by PARP10-mediated MARylation [7–10]. Activation of WNT signaling is an important driver of CRC development, and loss of MACROD2 function could thus contribute to CRC progression. Moreover, endogenous intracellular MACROD2 is recruited upon DNA damage and is able to reverse PARP1-mediated MARylation in the DNA-damage response [8].

In the present study, we examined the prognostic and predictive value of loss of MACROD2 protein expression in a series of 386 stage II and stage III clinically well-annotated primary colon cancers [11], and demonstrate that loss of MACROD2 protein expression is associated with poor survival in the subset of stage III colon cancer patients who were treated with adjuvant 5-FU-based chemotherapy.

## RESULTS

### MACROD2 expression and disease recurrence

**Figure 1:**
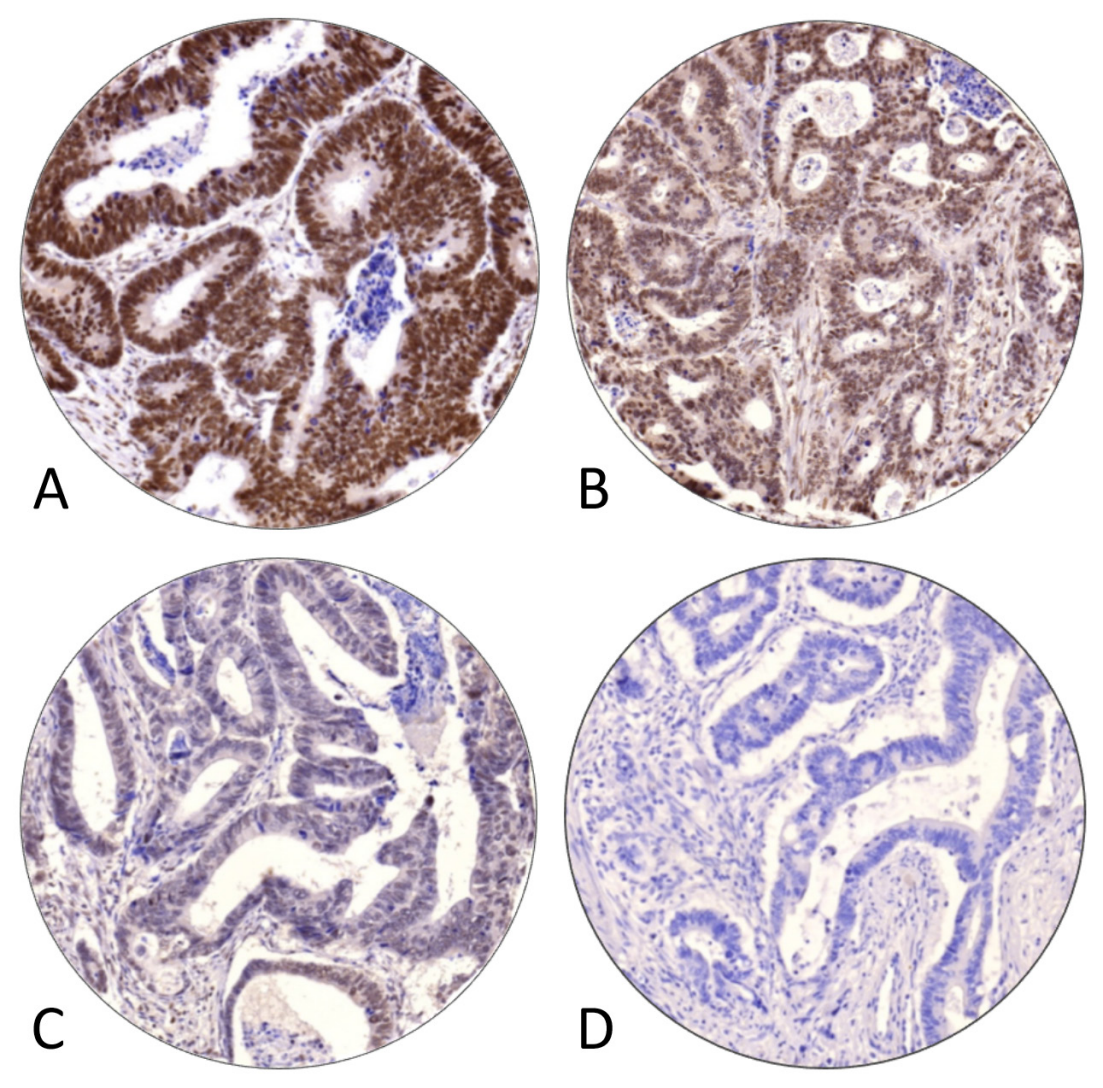
Representative examples of immunohistochemical staining intensities of MACROD2 expression, categories **(A)** ‘strong’, **(B)** ‘moderate’, **(C)** ‘weak’ and **(D)** ‘negative’, in stage II and III colon cancers.

Prognostic value of MACROD2 protein expression was examined by evaluation of immunohistochemical staining on TMAs that contained tissue biopsies from 226 stage II and 160 stage III colon cancers. Intensity of nuclear MACROD2 protein expression of epithelial cells could be scored for 343 patients (Figure 1) while 25 stage II and 18 stage III cases could not be evaluated due to technical reasons such as loss of cores from TMA slides. Dichotomization of the scores resulted in 180 tumors with low (52%) and 163 tumors with high (48%) nuclear MACROD2 expression of neoplastic cells. Baseline clinicopathological characteristics of these patients in relation to MACROD2 expression are presented in Table 1. MACROD2-low colon cancers were associated with higher N-stage (p=0.03, Table 1).

**Table 1:** Baseline clinicopathological characteristics of 343 stage II and III colon cancer patients with MACROD2-low and MACROD2-high expression

		Overall (n=343)	MACROD2-high (n=163)	MACROD2-low (n=180)	P-value
**Sex**	Male	183 (53.4)	82 (50.3)	101 (56.1)	0.33
	Female	160 (46.6)	81 (49.7)	79 (43.9)	
**Age (years)**	Mean (s.d.)	71.1 (11.9)	70.2 (13.1)	71.9 (10.7)	0.20
	Median (range)	73.2 (28.5-94.0)	73.9 (28.5-91.8)	72.8 (34.5-94.0)	
**Tumor location**	Right sided	152 (44.3)	69 (42.3)	83 (46.1)	0.55
	Left sided	191 (55.7)	94 (57.7)	97 (53.9)	
**Stage**	Stage II	201 (58.6)	102 (62.6)	99 (55.0)	0.19
	Stage III	142 (41.4)	61 (37.4)	81 (45.0)	
**Tumor size (mm)**	Mean (s.d.)	41.5 (19.1)	40.6 (17.3)	42.3 (20.7)	0.45
**Tumor stage**	T1	4 (1.2)	2 (1.2)	2 (1.1)	0.95
	T2	19 (5.5)	8 (4.9)	11 (6.1)	
	T3	289 (84.3)	139 (85.3)	150 (83.3)	
	T4	31 (9.0)	14 (8.6)	17 (9.4)	
**Nodal stage**	N0	201 (58.6)	102 (62.6)	99 (55.0)	**0.03**
	N1	97 (28.3)	48 (29.4)	49 (27.2)	
	N2	45 (13.1)	13 (8.0)	32 (17.8)	
**No. of nodes examined**	Mean (s.d.)	9.0 (5.2)	8.8 (5.1)	9.2 (5.3)	0.50
**Histological grade**	Well	20 (5.8)	10 (6.1)	10 (5.6)	0.83
	Moderate	274 (79.9)	128 (78.5)	146 (81.1)	
	Poor	49 (14.3)	25 (15.3)	24 (13.3)	
**Mucinous differentiation**	No	276 (80.5)	125 (76.7)	151 (83.9)	0.12
	Yes	67 (19.5)	38 (23.3)	29 (16.1)	
**Ulceration**	Absent	81 (23.6)	38 (23.3)	43 (23.9)	1.0
	Present	262 (76.4)	125 (76.7)	137 (76.1)	
**Angioinvasion**	Absent	278 (81.0)	137 (84.0)	141 (78.3)	0.23
	Present	65 (19.0)	26 (16.0)	39 (21.7)	
**Microsatellite stability status**	MSS	242 (70.6)	112 (68.7)	130 (72.2)	0.40
	MSI	56 (16.3)	30 (18.4)	26 (14.4)	
	*Unknown*	*45 (13.1)*	*21 (12.9)*	*24 (13.3)*	
**Type of surgery**	Emergency	46 (13.4)	23 (14.1)	23 (12.8)	0.84
**Perforation**	No	313 (91.3)	153 (93.9)	160 (88.9)	0.15
	Before surgery	15 (4.4)	6 (3.7)	9 (5.0)	
	During surgery	5 (1.5)	0 (0.0)	5 (2.8)	
	After surgery	10 (2.9)	4 (2.5)	6 (3.3)	
**Tumor spill**	No	333 (97.1)	159 (97.5)	174 (96.7)	0.87
	Yes	10 (2.9)	4 (2.5)	6 (3.3)	
**Adjuvant chemo**	No	237 (69.1)	116 (71.2)	121 (67.2)	0.50
	Yes	106 (30.9)	47 (28.8)	59 (32.8)	
**Recurrent disease**	No	234 (68.2)	119 (73.0)	115 (63.9)	0.09
	Yes	109 (31.8)	44 (27.0)	65 (36.1)	
**CRC mortality**		86 (25.1)	36 (22.1)	50 (27.8)	0.28
**Follow-up (months)**	Mean (s.d.)	61.0 (33.2)	64.4 (33.3)	57.9 (32.9)	0.07

Values in parentheses are percentages unless stated otherwise. P-values were calculated by chi-square tests or *t*-tests for continuous data. Bold p-values are considered significant (p<0.05).

**Figure 2:**
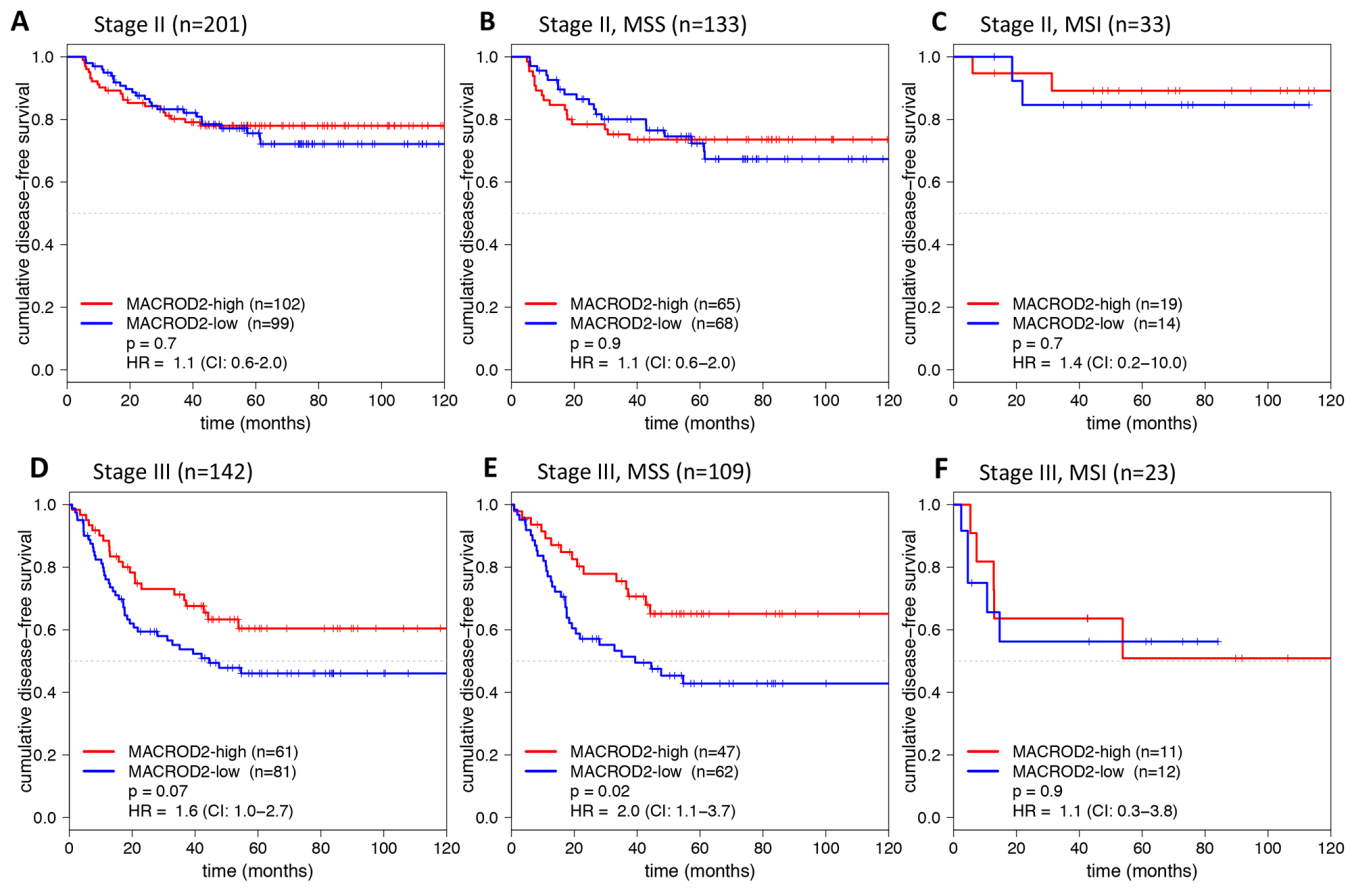
Kaplan-Meier plots for DFS (in months) stratified for MACROD2-high and MACROD2-low protein expression in stage II **(A-C)** and stage III **(D-F)** colon cancer patients including the subset of MSS (B, E) and MSI (C, F) patients. Log-rank p-values and Cox regression hazard ratios (HRs) with 95% confidence intervals (CIs) are reported.

MACROD2 expression was not associated with disease-free survival (DFS) in stage II colon cancers (Figure 2A). In stage III colon cancers, however, low expression of MACROD2 showed a poorer DFS than high expression of MACROD2, although this difference did not reach statistical significance (p=0.07; HR=1.6, 95% CI 1.0-2.7; Figure 2D). Stratification by MSI status (Table 1) showed that in microsatellite stable (MSS) stage III colon cancers (n=109) low expression of MACROD2 was associated with poor DFS (p=0.02; HR=2.0, 95% CI 1.1-3.7; Figure 2E). This effect was not observed in MSS stage II colon cancers (n=133; p=0.9; Figure 2B). The limited numbers of MSI stage II (n=33) and stage III (n=23) samples did not allow for meaningful comparison of DFS in MACROD2-low *versus* MACROD2-high MSI colon cancers (Figure 2C, 2F).

### MACROD2 expression and response to 5-FU-based adjuvant chemotherapy

**Figure 3:**
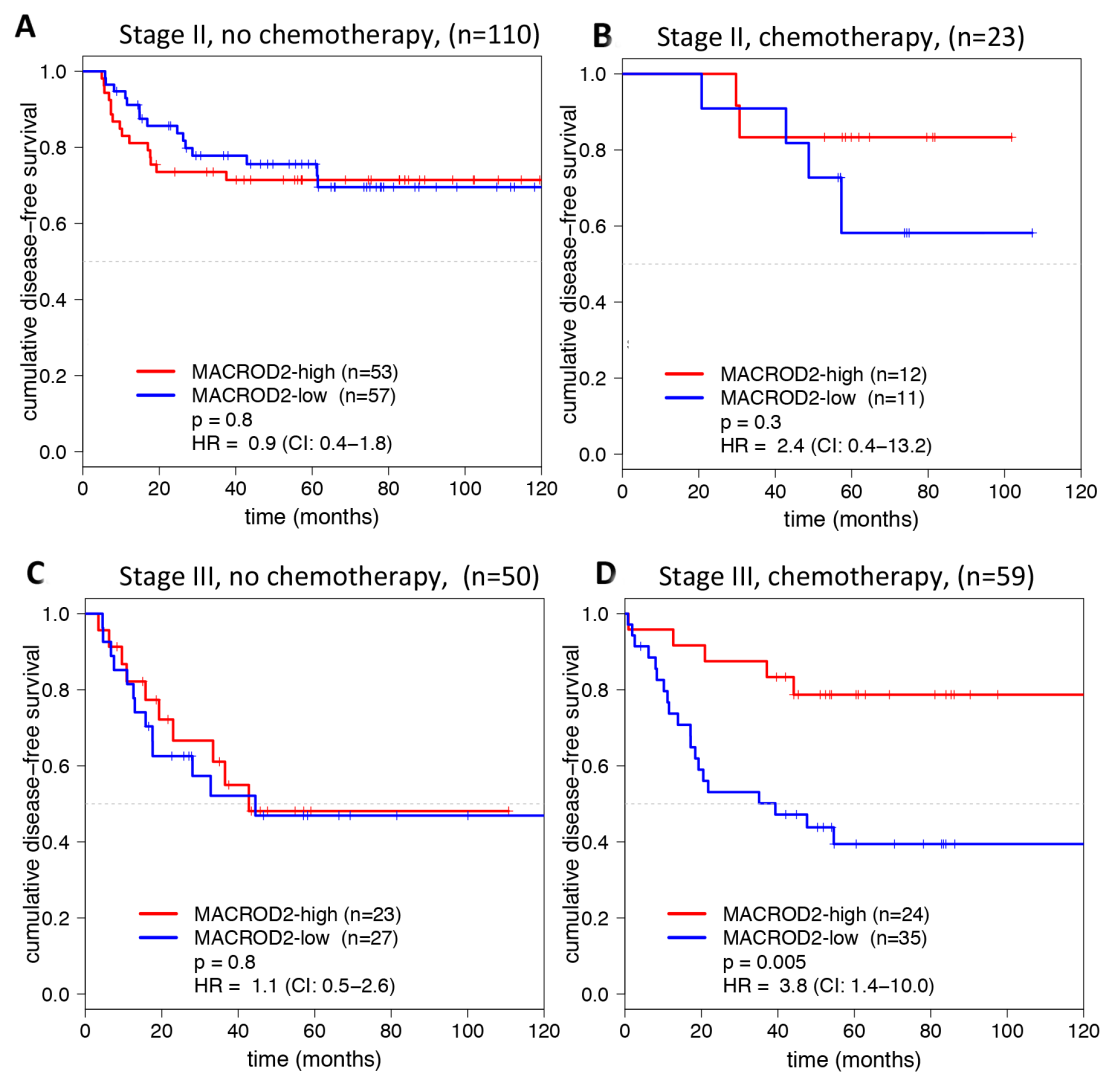
DFS curves (in months) for MACROD2 expression in MSS colon cancer patients who did not receive **(A, C)** and did receive **(B, D)** 5-FU-based adjuvant chemotherapy for stage II (A, B) and stage III (C, D) colon cancer patients. Log-rank p-values and Cox regression HRs (95% CI) are reported.

Adjuvant chemotherapy could influence the prognostic effect of MACROD2 protein expression. Therefore, this parameter was used for further stratification. In total 23 of 133 MSS stage II and 59 of 109 MSS stage III colon cancer patients were treated with 5-fluorouracil (5-FU)-based adjuvant chemotherapy. No effect of MACROD2 expression on DFS was observed within the subgroups that did not receive adjuvant chemotherapy (Figure 3A, 3C). However, loss of MACROD2 protein expression was strongly associated with poor DFS in MSS stage III colon cancer patients that did receive 5-FU-based adjuvant chemotherapy (p=0.005; HR=3.8, 95% CI 1.4-10.0; Figure 3D). The same tendency was observed for MSS stage II colon tumors, although the number of samples was too small to draw definitive conclusions (n=23; p=0.3; HR=2.4, 95% CI 0.4-13.2; Figure 3B).

### Multivariate analysis

Association of MACROD2 expression with DFS was tested by a multivariate model that included established clinicopathological parameters. This multivariate model showed that MACROD2 expression was not an independent prognostic factor in the entire study population (data not shown). However, since MACROD2-low expression was associated with poor DFS in the subgroup of stage III colon cancer patients who received 5-FU-based adjuvant chemotherapy, two separate models were built for stage II and stage III colon cancers. While MACROD2 expression was not retained by the model in stage II colon cancers, MACROD2 expression was retained in the multivariate model for stage III colon cancers in addition to ‘tumor location’, ‘T-stage’, ‘angioinvasion’ and ‘perforation’ (Table 2).

**Table 2:** Clinicopathological parameters that retained in a multivariate stepwise backward Cox-regression model (p<0.05) of stage III colon cancers

Parameter	HR (95% CI)	P-value
**MACROD2 expression**	0.6 (0.3-1.0)	0.046
**Tumor location**	1.8 (1.0-3.1)	0.041
**T-stage**	1.8 (1.0-3.1)	0.038
**Angioinvasion**	2.5 (1.4-4.2)	0.001
**Perforation**	1.4 (1.0-2.0)	0.042

## DISCUSSION

The current study showed that low MACROD2 protein expression was associated with poor DFS in stage III MSS colon cancer patients who received 5-FU-based adjuvant chemotherapy (Figure 3D). The observation that MACROD2 expression was predictive in colon cancers treated for 5-FU-based adjuvant therapy (Figure 3) may suggest that the underlying biological mechanism is involvement of MACROD2 in DNA damage response, which is one of the known functions of MACROD2 [8]. It has previously been demonstrated that MACROD2 is involved in DNA damage signaling and is capable to reverse PARP1-mediated MARylation [8]. Notably, presence of MACROD2 could effectuate suppression of PARP1 activity [8], which is involved in DNA repair of incorporated 5-FU and its metabolites in the genome that exacerbates replication stress [12]. Consequently, tumor cells having low protein expression of MACROD2 may effectively enable PARP1-dependent DNA repair. Thus, one could speculate that 5-FU treatment combined with a small molecule PARP inhibitor may be lethal for tumor cells that have MACROD2-low protein expression. One *in vitro* study showed that PARP inhibition synergizes with FdUrd, which is a metabolite of 5-FU, in MSI and MSS colon cancer cells [13].

*MACROD2* has been identified to be the most frequent recurrent breakpoint gene in advanced CRC, which was observed in more than 40% of cases [6]. Accordingly, MACROD2 was a candidate biomarker to further examine its prognostic and predictive value. The present study demonstrated that low MACROD2 protein expression was associated with poor DFS, which is concordant with the hypothesis that SV breakpoints in *MACROD2* cause loss of normal gene function. However, we were not able to correlate SV breakpoints in *MACROD2* to loss of nuclear staining because all *MACROD2* breakpoints are located downstream of the first three exons that encode the epitope of the polyclonal antibody that was used for immunohistochemical staining (data not shown).

Although this study comprised a large retrospective cohort of 343 stage II and stage III colon cancer patients with well-documented clinical information, the sample size was insufficient to extensively test interactions of MACROD2 expression with other clinicopathological parameters. Furthermore, validation of the predictive value of MACROD2 expression for response to 5-FU-based therapy is required in a large independent prospective randomized clinical trial minimizing bias that might be introduced by unknown confounding factors associated with DFS rates and MACROD2 expression in the current study. In addition, as currently adjuvant treatment regimens are used other than 5-FU-based monotherapy, also the predictive effect of MACROD2 on 5-FU in combination with other chemotherapeutic agents such as irinotecan or oxaliplatin needs to be examined. It is unclear how MACROD2 expression may be associated with clinical outcome in relation to drug responsiveness, exemplified by a primary breast cancer study that showed that MACROD2 overexpression was associated with worse survival, probably due to resistance to anti-estrogen receptor-alpha therapy (tamoxifen) [14].

In conclusion, loss of nuclear MACROD2 protein expression predicts poor response to adjuvant 5-FU-based chemotherapy in MSS stage III colon cancers. Further studies are warranted to validate this potential biomarker to stratify colon cancer patients for response to 5-FU-based chemotherapy in the clinic and to dissect the putative essential function of MACROD2 with respect to therapy resistance.

## MATERIALS AND METHODS

### MACROD2 immunohistochemistry using tissue microarrays

Archival formalin-fixed and paraffin-embedded (FFPE) material from 226 stage II and 160 stage III colon cancers was used to construct tissue microarrays (TMAs) as described by Belt *et al.* [11]. Microsatellite instability (MSI) status has previously been determined using a DNA-based test [11]. Tumor specimens and matched clinical data were obtained in compliance with the ‘Code for Proper Secondary Use of Human Tissue in The Netherlands’ (https://www.federa.org/). A detailed overview of clinicopathological characteristics is given in Table 1.

Four μm sections of TMAs were mounted on glass slides, deparaffinized by xylene and rehydrated with a decreasing alcohol series. Staining for MACROD2 was performed upon antigen retrieval by microwave heating in citric acid (10 mM, pH6.0) and endogenous peroxidase neutralization in 0.3% hydrogen peroxide in methanol for 25 minutes. The primary rabbit polyclonal antibody directed against human MACROD2 (HPA049076; Atlas Antibodies AB, Stockholm, Sweden) was incubated one hour at a 1:175 dilution at room temperature, followed by incubation with polymer labeling for 30 minutes at room temperature (BrightVision, Immunologic, Duiven, The Nederlands). Secondary antibodies were visualized by liquid diaminobenzidine (DAB) substrate chromogen system. Slides were counterstained with Mayer's haematoxylin. Staining of FFPE normal kidney tissue was used as a positive control and incubation without primary antibody as a negative control.

### Evaluation of MACROD2 protein expression

Immunohistochemical stainings were digitally captured as previously described [11]. Individual TMA core biopsies were scored for intensity of nuclear MACROD2 protein expression of neoplastic epithelial cells (categories: negative, weak, moderate, strong; Figure 1) using dedicated TMA scoring software (v1.15.2, 3DHISTECH Ltd., Budapest, Hungary). TMA cores that contained less than 30% intact (epithelial) tumor tissue were considered non-representative and excluded. TMA-cores from 56 tumors were evaluated by an independent observer (NTCvG) to assess inter-observer agreement for lowest MACROD2 intensity, which Cohen's weighted kappa score was K_w_=0.6 [15, 16]. Intensity scores from tumor central and peripheral core biopsies [11, 17] were similar (Wilcoxon signed rank test, p=0.97).

Protein expression scores for MACROD2 were dichotomized for analysis of patient subgroups. First, the data was randomly split into five subsets. Next, the optimal cut-off for dichotomizing scores into a high- or low-expression group was based on 4/5th of the dataset using Receiver Operating Characteristic (ROC) curve analysis for survival data with 5-year DFS as the outcome of interest. This procedure was repeated five times, with 1/5th of the dataset varying. The final cut-off was the cut-off that was most often selected. In this way, the optimal cutoff for MACROD2 was set to ‘low expression’ for negative, weak and moderate intensity scores and ‘high expression’ for strong intensity scores. Optimal cutoff for MACROD2 was identical for all five iterations.

### Statistical analysis

Statistical analysis was performed in R (version 3.2.2). Differences in baseline clinicopathological characteristics between patients with MACROD2-high and MACROD2-low protein expression were analyzed using Chi-square or student's *t*-tests. Univariate associations between DFS and MACROD2 protein expression was evaluated by Kaplan-Meier analysis. Cumulative survival rates were visualized by Kaplan-Meier curves (displayed for 120 months) and compared using a two-sided log-rank test (univariate). Hazard Ratios (HR) for MACROD2 expression were calculated using Cox regression analysis. Associations of DFS and known prognostic clinicopathological parameters were evaluated by multivariate Cox's proportional hazards regression analysis using stepwise backward elimination. Input parameters in addition to MACROD2 expression were tumor stage, T- and N-stage, isolated tumor deposits, MSI status, tumor location (right sided), angioinvasion, histological grade, ulceration, perforation, and tumor spill [18–21]. This analysis was also performed by stratification for tumor stage. P-values less than 0.05 were considered significant.
